# scGPA: an LLM-assisted workflow for directional virtual gene perturbation analysis from single-cell transcriptomes

**DOI:** 10.1186/s12864-026-13270-0

**Published:** 2026-08-25

**Authors:** Haijun Tang, Hening Li, Qinghao Zhao, Xiang Zhou, Lei Peng, Yangjie Cai, Rongzhen Lin, Yufeng Li, Yiyi Yuan, Wenyu Feng, Yun Liu, Zezheng Liu, Qingchu Li

**Affiliations:** 1 Department of Spine Surgery, Academy of Orthopedics, Orthopaedic Hospital of Guangdong Province, The Third Affiliated Hospital of Southern Medical University, Guangzhou, China; 2 Department of Oncology, The First Affiliated Hospital of Guangxi Medical University, Nanning, Guangxi China; 3 Department of Spine and Osteopathic Surgery, The First Affiliated Hospital of Guangxi Medical University, Nanning, Guangxi China; 4 Department of Bone and Joint Surgery and Sports medicine, The Second Affiliated Hospital of Guangxi Medical University, Nanning, China

**Keywords:** Artificial intelligence, Large language model, Virtual cell, Single-cell RNA sequencing

## Abstract

**Background:**

Existing virtual perturbation methods can often infer directional changes by comparing predicted post-perturbation expression profiles with control cells. However, workflows that directly return direction-specific downstream candidate genes together with confidence scores, evidence support and interpretable summaries remain limited. We developed scGPA, an LLM-assisted workflow system for directional single-cell virtual gene perturbation analysis.

**Methods:**

scGPA starts from raw single-cell RNA sequencing data and performs quality control, normalization, dimensionality reduction, clustering and cell-group selection. It then constructs cell-group-specific wild-type regulatory networks using repeated subsampling, principal component regression (PCR)/Ridge-based network inference and CP tensor denoising. Based on these networks, scGPA simulates dose-aware virtual knockdown of the target gene and applies signed perturbation propagation to estimate the magnitude and direction of downstream transcriptional responses. LLM assistance is used for marker-based cell-type annotation, evidence-guided candidate prioritization and user-facing biological summarization.

**Results:**

We benchmarked scGPA across five public Perturb-seq datasets and compared its performance with GEARS, scGPT and a random baseline. The overall correct prediction rate of scGPA was 23.0%, exceeding those of GEARS (20.7%), scGPT (15.1%) and the random baseline (13.6%). These results indicate that scGPA achieved a higher correct prediction rate than the two comparator models and the random baseline. We subsequently evaluated scGPA using a public osteosarcoma single-cell dataset and performed qRT-PCR validation in 143B osteosarcoma cells. Among genes with significant experimental changes, scGPA achieved a directional concordance of 76.9%. When all tested downstream genes were counted, 37.0% were directionally correct, 51.9% showed no significant change and 11.1% changed in the opposite direction.

**Conclusions:**

scGPA provides a practical workflow system for predicting and prioritizing direction-specific downstream transcriptional responses after target-gene perturbation. By integrating single-cell regulatory network inference, signed virtual perturbation and LLM-assisted interpretation, scGPA supports target-gene function inference and downstream mechanistic investigation from single-cell transcriptomic data.

**Supplementary Information:**

The online version contains supplementary material available at https://doi.org/10.1186/s12864-026-13270-0.

## Introduction

Virtual perturbation models are computational frameworks designed to simulate and predict how gene knockout, gene overexpression, drug exposure, environmental stimuli, or disease progression alter cellular states before direct wet-lab validation. Their central objective is to establish a computable, interpretable, and extrapolatable mapping between high-dimensional molecular phenotypes and cell-state transitions [1]. In the single-cell era, the concept of virtual perturbation has evolved from static analyses based primarily on differential expression or individual pathways into a broader framework for modelling transcriptional regulation, cell-state trajectories, drug sensitivity, cell-cell communication, and population-level distributional changes under perturbation [2]. These models are valuable at three levels. First, they enable high-throughput in silico screening when experimental resources are limited but the number of candidate genes or compounds is large, thereby reducing the cost of blind experimentation. Second, by predicting the magnitude, direction, and potential mechanisms of perturbation-induced changes, they provide a principled basis for studying disease progression, identifying functional genes, and prioritizing therapeutic targets [3–6]. Third, they support a simulation-to-validation closed loop in which hypotheses are first generated digitally and then tested experimentally through CRISPR assays, organoids, organ-on-chip systems, or related platforms, thereby shifting precision medicine and drug discovery from an experience-driven to a prediction-driven paradigm [3, 6–8]. With the rise of Perturb-seq, CROP-seq, ECCITE-seq, Perturb-ATAC, and related perturbational single-cell technologies, virtual perturbation studies have entered a high-throughput, multimodal, and cross-scale phase [9–13]. At the same time, the introduction of machine learning, deep learning, graph neural networks, and foundation models has increasingly enabled perturbation modelling to move beyond describing known perturbation outcomes toward predicting unobserved perturbational scenarios [14]. The integration of AI with virtual perturbation modelling provides clear advantages: AI can uncover latent structure and higher-order interactions from sparse, noisy, and nonlinear single-cell datasets that are difficult to model with classical statistical frameworks, while perturbational biology provides biologically meaningful supervision targets centered on gene regulation, drug response, and cell-state transitions [15]. More importantly, with the emergence of large language models and LLM-assisted workflow systems, AI is no longer restricted to numerical prediction alone, but is increasingly involved in cell-type naming, mechanistic prioritization, literature-grounded evidence checking, and multi-source knowledge integration, thereby transforming virtual perturbation tools into interactive, explainable, and verifiable research workflows [16, 17].

At present, the field of virtual perturbation has developed several major methodological families. The first comprises statistical inference and shallow machine-learning approaches, such as scMAGeCK [18], Augur [19], scDist [20], Milo [21], Mixscape [22], and CellDrift [23], which are particularly strong in differential abundance analysis, cell-state discrimination, temporal profiling, and confounder control, and thus provide robust local inference in well-defined tasks. The second family consists of perturbation-similarity or pharmacogenomic latent-space models, such as CaDRReS-Sc, which integrate drug knowledge bases and expression features to prioritize drug responses and combinatorial therapies [24, 25]. The third family includes gene-regulatory-network-prioritizing models, including CellOracle [26, 27], SCENIC+ [28], SCING [29], RENGE [30], and related digital knockout models, which explicitly or implicitly model regulatory graphs and are therefore more biologically grounded for in silico gene knockout, transcription-factor perturbation, and cell-state transition analysis. The fourth and rapidly emerging family consists of foundation models, including Geneformer [17], scGPT [31], scELMo [32], and scFoundation [33], which are pretrained on tens of millions of single cells and are beginning to shift perturbation modelling from task-specific prediction to more generalizable single-cell representation learning.

Despite their rapid development, current mainstream models share several limitations. First, several recent perturbation-response models, including GEARS, scGPT and CPA, can predict post-perturbation gene-expression profiles, and gene-level up- or down-regulation can be inferred by comparing predicted expression with control cells [34]. However, these predicted directions are often derived indirectly as a post hoc difference between predicted perturbed and control expression states. Such expression-difference-based direction calling may be affected by baseline expression estimation, systematic variation and model-specific normalization, and it does not explicitly represent how the attenuated regulatory output of a target gene propagates through signed regulatory relationships. Second, a considerable fraction of existing methods focus on extrapolating from already observed perturbation datasets and are less suited for constructing an interpretable virtual knockout framework directly from wild-type single-cell expression alone. Third, most tools function as isolated prediction engines that output ranked gene lists but offer limited support for stability assessment, evidence verification, or natural-language interpretation, which restricts their utility in experimental design.

To address these gaps, we developed scGPA, an LLM-assisted virtual perturbation workflow system for single-cell data, with DoseDirKO as its core perturbation engine. The novelty of this work is that scGPA infers a cell-group-specific wild-type regulatory network, attenuates the outgoing effects of the target gene and propagates the perturbation signal through signed regulatory edges to generate a signed response vector. Therefore, direction is estimated from regulatory propagation rather than only from post hoc comparison of predicted expression profiles. scGPA further integrates LLM-assisted cell-type annotation, evidence retrieval, candidate prioritization and natural-language reporting, producing ranked “Top Up” and “Top Down” outputs with scores, confidence values and biological summaries.

## Results

### scGPA analytical workflow

**Fig. 1:**
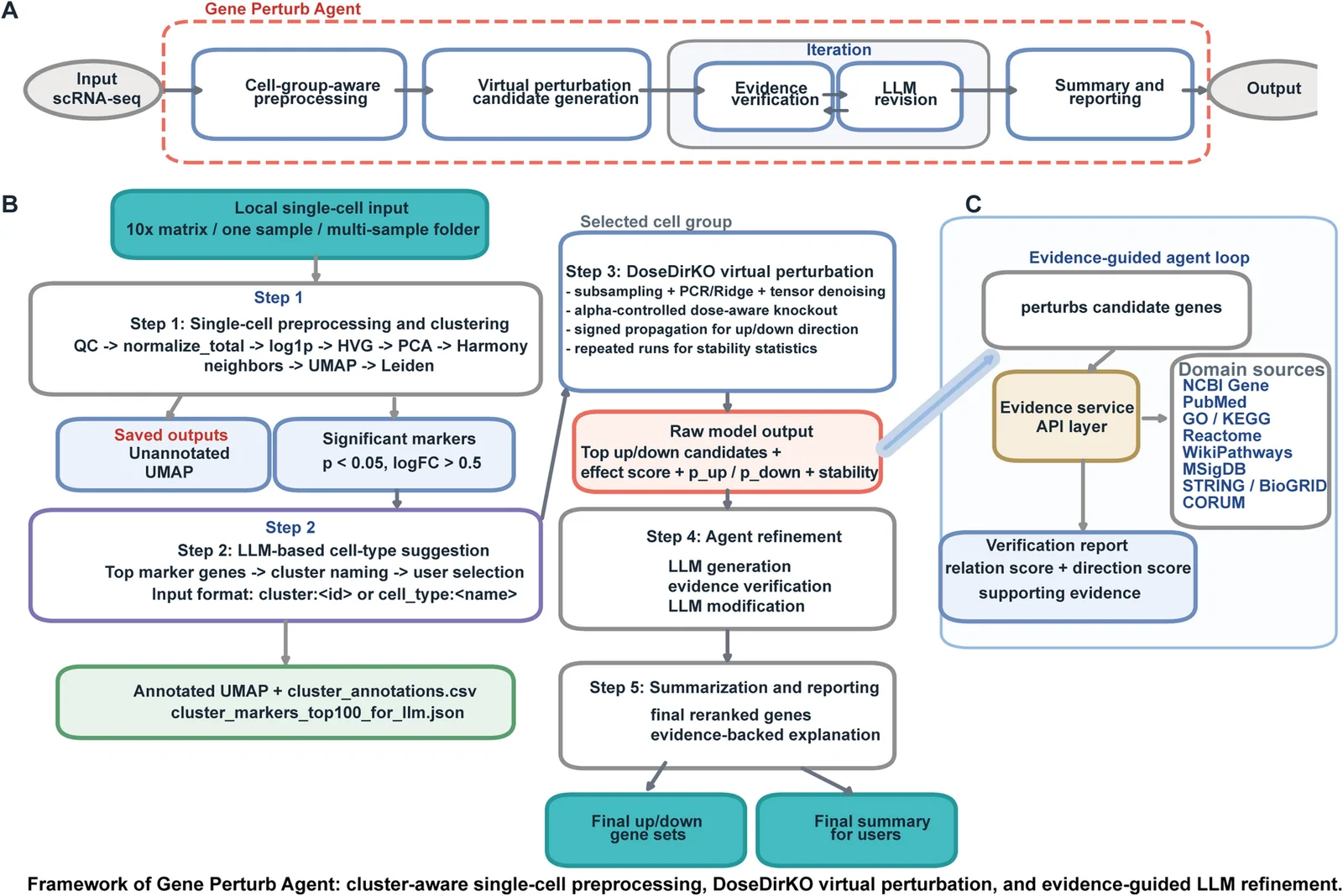
Framework of scGPA for single-cell virtual perturbation analysis. **A **Overview of scGPA, consisting of cell-group-aware preprocessing, virtual perturbation candidate generation, evidence-guided iteration and summarization. Evidence verification is iterated with LLM revision before final reporting. **B** Workflow of scGPA. The DoseDirKO module infers a wild-type regulatory network by subsampling, PCR/Ridge-based network construction and tensor denoising, and simulates dose-aware knockout of a target gene through the parameter alpha. Signed propagation and repeated runs are used to derive directional effects, effect scores and stability statistics. The downstream refinement module performs LLM-assisted generation, evidence verification, LLM revision and summarization to produce the final ranked output and explanatory report. **C** Schematic of the evidence-guided refinement loop. Candidate claims derived from the perturbation output are evaluated against external domain resources, including NCBI Gene, PubMed, GO, Reactome, KEGG, WikiPathways, MSigDB Hallmark, STRING, BioGRID and CORUM. The resulting verification report is used to retain, rerank or revise candidate claims before final reporting

scGPA enables cluster-aware single-cell virtual perturbation analysis by coupling a network-based perturbation engine with an evidence-guided LLM refinement loop (Fig. 1A and Methods). Starting from a local single-cell dataset in 10x matrix format, scGPA performs standardized preprocessing, including cell-level quality control, library-size normalization, log transformation, highly variable gene selection, principal component analysis, Harmony integration for multi-sample inputs, neighborhood graph construction, UMAP embedding and Leiden clustering. Marker genes are then extracted for each cluster, and an LLM-assisted annotation step proposes candidate cell identities. After the user selects a target cluster or cell type, scGPA initiates the DoseDirKO module to simulate perturbation of a user-specified gene within the selected cellular context.

Specifically, DoseDirKO first infers a wild-type regulatory network from the selected cell group using repeated subsampling, PCR/Ridge-based network construction and tensor denoising. It then applies dose-aware virtual knockout through the parameter alpha and estimates downstream responses by combining network displacement with signed propagation. The raw output contains preliminary lists of upregulated and downregulated candidate genes together with effect scores, directional probabilities and stability statistics aggregated across repeated runs (Fig. 1B). These candidates are subsequently passed to the evidence-guided refinement loop (Fig. 1C), in which candidate claims are formulated by the LLM and evaluated against external biomedical resources, including NCBI Gene, PubMed, GO, Reactome, KEGG, WikiPathways, MSigDB Hallmark, STRING, BioGRID and CORUM. The evidence service retrieves relevant records through Web APIs and compiles a verification report containing relation and direction scores together with supporting evidence for each candidate interaction.

Based on the verification report, the LLM revision module retains, reranks or modifies candidate claims before a final summarization step generates an evidence-backed report for the selected cell group. This design separates mechanistic prediction from knowledge-guided refinement: the single-cell perturbation engine provides cluster-specific candidate responses, whereas the refinement loop evaluates their biological plausibility and improves interpretability before reporting the final top upregulated and downregulated genes. Because the single-cell matrix is processed locally and only compact intermediate representations are used during the refinement stage, the framework preserves sample-level privacy while producing an experimentally actionable output for downstream validation.

### Benchmarking of scGPA on public Perturb-seq datasets

To further evaluate the generalizability of scGPA, we benchmarked its directional prediction performance across five public Perturb-seq datasets: DixitRegev2016_K562_TFs_7_days, PapalexiSatija2021_eccite_RNA, NormanWeissman2019, FrangiehIzar2021_RNA and AdamsonWeissman2016_10 × 010. A total of 304 perturbation targets were included, comprising 9, 20, 64, 156 and 55 targets from the respective datasets. scGPA was compared with GEARS, scGPT and an empirical random baseline. For each target, each model selected the top 10 predicted upregulated genes and the top 10 predicted downregulated genes. A prediction was considered correct only when its direction matched a significant transcriptional change observed experimentally.

**Fig. 2:**
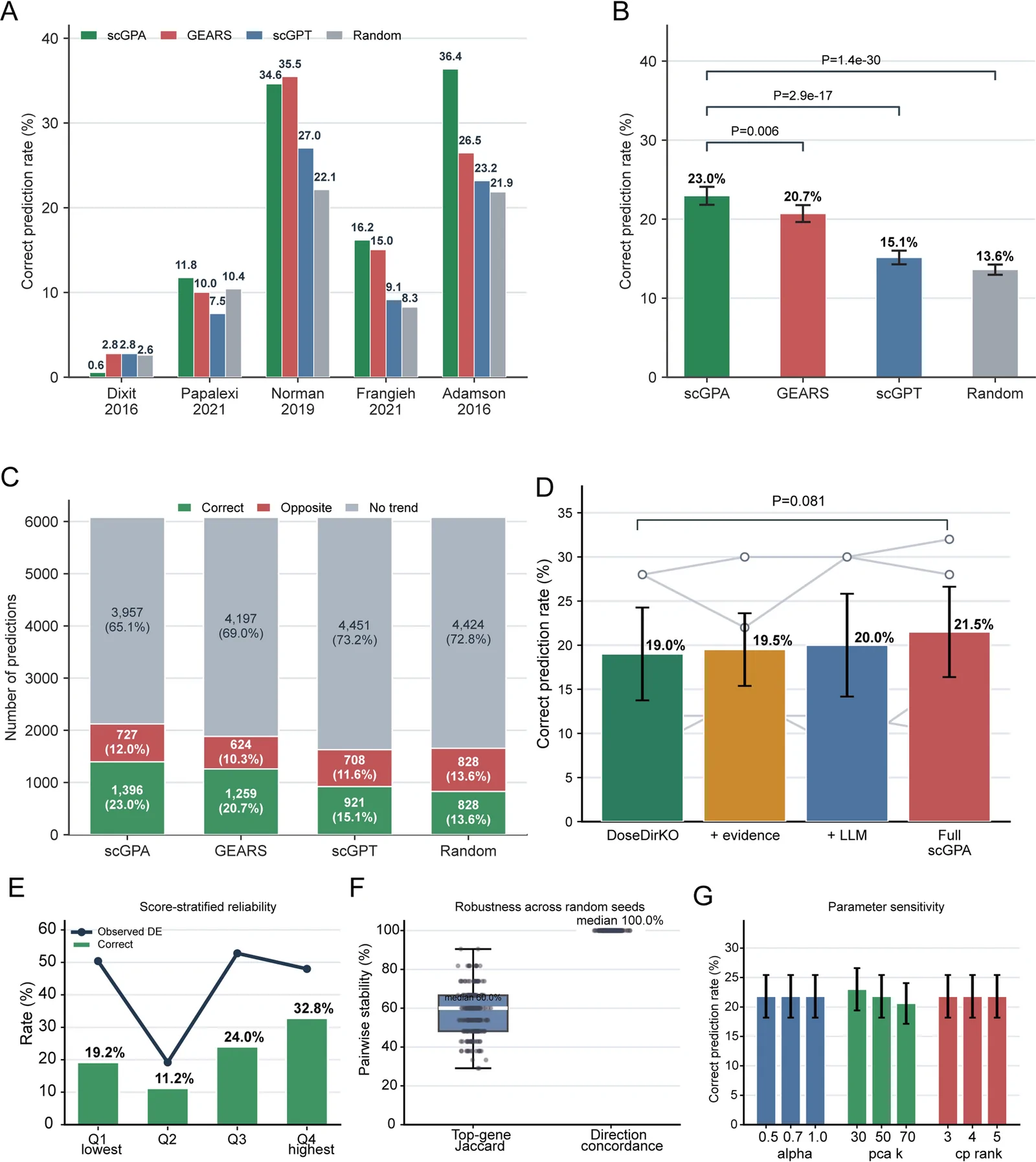
Benchmarking, ablation and robustness evaluation of scGPA on public Perturb-seq datasets. **A** Dataset-level correct prediction rates across five Perturb-seq datasets: DixitRegev2016_K562_TFs_7_days, PapalexiSatija2021_eccite_RNA, NormanWeissman2019, FrangiehIzar2021_RNA and AdamsonWeissman2016_10 × 010. **B** Mean target-level correct prediction rates across the 304 matched perturbation targets. Bars and error bars represent the mean and s.e.m., respectively. P values were calculated using one-sided paired Wilcoxon signed-rank tests. **C** Numbers and proportions of the 6,080 predictions from each method classified as correct, opposite or showing no significant trend. **D** Ablation analysis of the scGPA workflow. **E** Score-stratified reliability analysis of scGPA predictions. **F** Robustness of scGPA predictions across random seeds. DoseDirKO was repeatedly run using different random seeds. **G** Parameter sensitivity analysis. Correct prediction rates are shown after varying perturbation strength (alpha = 0.5, 0.7 and 1.0), PCA dimensionality (pca_k = 30, 50 and 70) and CP tensor rank (cp_rank = 3, 4 and 5). Bars show mean correct prediction rates, and error bars indicate the standard error of the mean

At the dataset level, scGPA achieved higher correct prediction rates than GEARS in the Papalexi, Frangieh and Adamson datasets and outperformed scGPT in four of the five datasets, with Dixit being the exception (Fig. 2A). Across all 304 targets, the mean correct prediction rates of scGPA, GEARS, scGPT and the random baseline were 23.0%, 20.7%, 15.1% and 13.6%, respectively (Fig. 2B). Target-level comparisons using one-sided paired Wilcoxon signed-rank tests showed that scGPA achieved significantly higher correct prediction rates than GEARS (*P* = 0.006), scGPT (*P* = 2.9 × 10⁻¹⁷) and the random baseline (*P* = 1.4 × 10⁻³⁰). Among the 6,080 predictions generated by each method, scGPA yielded 1,396 correct predictions (23.0%), 727 predictions in the opposite direction (12.0%) and 3,957 predictions with no significant trend (65.1%). The corresponding numbers for GEARS were 1,259 (20.7%), 624 (10.3%) and 4,197 (69.0%), whereas those for scGPT were 921 (15.1%), 708 (11.6%) and 4,451 (73.2%), respectively (Fig. 2C). The random baseline achieved a mean correct prediction rate of 13.6% across 1,000 repetitions. Collectively, these results demonstrate that scGPA provides significantly improved directional prediction of downstream transcriptional responses compared with GEARS, scGPT and the random baseline across diverse Perturb-seq datasets.

### Ablation analysis distinguishes the prediction module from downstream refinement

To determine whether the benchmark performance of scGPA was driven by the core perturbation model or by downstream knowledge-guided refinement, we performed an ablation analysis using a fixed subset of 20 perturbation targets from four public Perturb-seq datasets, with five targets evaluated per dataset.

DoseDirKO alone achieved a mean correct prediction rate of 19.0% across the four datasets. Adding evidence verification slightly increased the correct prediction rate to 19.5%, while adding LLM refinement increased it to 20.0%. The full scGPA pipeline achieved the highest mean correct prediction rate of 21.5% (Fig. 2D). Although the improvement of the full pipeline over DoseDirKO alone did not reach statistical significance in this small benchmark set (paired Wilcoxon signed-rank test, *P* = 0.081), the ablation results indicate that DoseDirKO provides the core directional prediction signal, whereas evidence verification and LLM refinement mainly contribute to candidate prioritization, evidence traceability and user-facing biological interpretation.

### Reliability and robustness analysis of scGPA predictions

We further evaluated the reliability and robustness of scGPA predictions. We first assessed whether the model score could be used for candidate-gene prioritization. Predicted genes were divided into four quartiles according to the absolute value of the signed perturbation score. The highest-score group showed the highest correct prediction rate (32.8%), whereas the correct prediction rates in the first, second and third quartiles were 19.2%, 11.2% and 24.0%, respectively (Fig. 2E). These results indicate that the score can, to some extent, reflect the accuracy of predicted genes. Genes with higher scores are more likely to be correct and are therefore more suitable for prioritization in downstream experimental validation.

We next evaluated the robustness of the model to sampling variability by repeating DoseDirKO with five different random seeds. Across repeated runs with different random seeds, the median pairwise Jaccard index of the top-ranked predicted gene sets was 60.0%, indicating moderate overlap among top candidate genes. More importantly, the median directional concordance among overlapping genes was 100.0% (Fig. 2F). These results suggest that different random seeds may affect whether some borderline candidate genes enter the top list, but for repeatedly selected high-confidence candidate genes, the directional predictions of scGPA are highly stable.

Finally, we evaluated the sensitivity of the model to key parameters, including perturbation strength (α = 0.5, 0.7 and 1.0), PCA dimensionality (pca_k = 30, 50 and 70) and CP tensor rank (cp_rank = 3, 4 and 5). Across these parameter settings, the mean correct prediction rate ranged from 20.6% to 23.0%. Compared with the default setting, the mean top-gene Jaccard index was 84.6%, and the mean directional concordance was 99.96% (Fig. 2G). These results indicate that scGPA is robust to changes in key parameters. 

### scGPA predicted target-specific downstream transcriptional responses in osteosarcoma cells

**Fig. 3:**
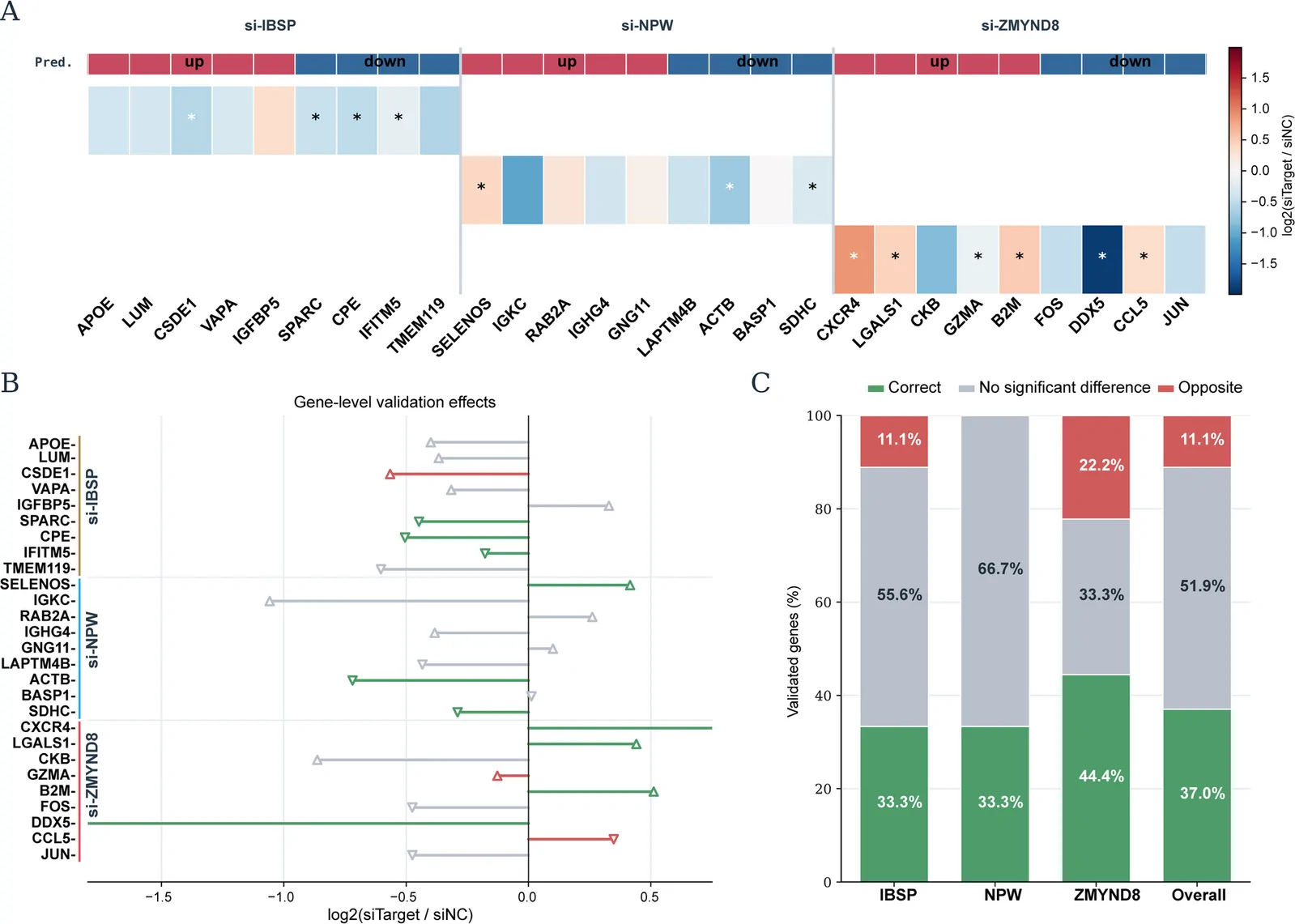
Experimental validation of scGPA-predicted downstream genes in osteosarcoma cells. **A** Heatmap of qRT-PCR-measured expression changes after silencing IBSP, NPW or ZMYND8 in 143B cells. Color indicates log2(siTarget/siNC), and the top bar indicates the predicted direction. **B** Gene-level validation effects. Horizontal lines show log2(siTarget/siNC); triangles indicate predicted direction. Green, correct; gray, no significant difference; red, opposite. **C** Proportions of correct, no significant difference and opposite predictions for each target gene and overall. *: *P* < 0.05

Based on the scGPA prediction results (Table 1), we selected top-ranked downstream candidates for experimental validation in 143B osteosarcoma cells after silencing IBSP, NPW and ZMYND8. For each target gene, five predicted upregulated genes and four predicted downregulated genes were subjected to qRT-PCR analysis. As shown in Fig. 3A and B, the experimentally measured expression changes showed target-dependent agreement with the directions predicted by scGPA.

**Table 1:** scGPA-predicted results for osteosarcoma cells

Target gene	Direction	Predicted gene	Score	Confidence
IBSP	Top Up	APOE	0.310	0.600
		LUM	0.270	0.600
		CSDE1	0.140	0.200
		VAPA	0.100	0.200
		IGFBP5	0.080	0.200
	Top Down	IBSP	0.370	0.800
		SPARC	0.330	0.800
		CPE	0.260	0.600
		IFITM5	0.230	0.600
		TMEM119	0.100	0.200
NPW	Top Up	SELENOS	0.160	0.200
		IGKC	0.140	0.200
		RAB2A	0.120	0.200
		IGHG4	0.110	0.200
		GNG11	0.100	0.200
	Top Down	NPW	0.340	0.800
		LAPTM4B	0.140	0.200
		ACTB	0.120	0.200
		BASP1	0.100	0.200
		SDHC	0.080	0.200
ZMYND8	Top Up	CXCR4	0.160	0.200
		LGALS1	0.140	0.200
		CKB	0.120	0.200
		GZMA	0.100	0.200
		B2M	0.080	0.200
	Top Down	ZMYND8	0.370	0.800
		FOS	0.200	0.600
		DDX5	0.140	0.200
		CCL5	0.130	0.200
		JUN	0.120	0.200

Following IBSP silencing, three predicted downregulated genes, SPARC, CPE and IFITM5, were significantly decreased in the expected direction. Among the predicted upregulated genes, IGFBP5 showed a nonsignificant increase, whereas APOE, LUM and VAPA showed no significant directional change. CSDE1 was significantly decreased, opposite to the predicted upregulation. Overall, 3 of 9 IBSP-associated downstream candidates were directionally correct, 5 showed no significant difference, and 1 changed in the opposite direction.

After NPW silencing, SELENOS was significantly upregulated as predicted, whereas ACTB and SDHC were significantly downregulated in the expected direction. The remaining predicted candidates, including IGKC, RAB2A, IGHG4, GNG11, LAPTM4B and BASP1, did not show significant changes. No NPW-associated candidate showed a significant opposite response. Thus, 3 of 9 NPW-associated downstream candidates were directionally correct, and 6 showed no significant difference.

In the ZMYND8 knockdown group, CXCR4, LGALS1 and B2M were significantly upregulated as predicted, and DDX5 was significantly downregulated in the expected direction. By contrast, GZMA and CCL5 changed in the opposite direction, whereas CKB, FOS and JUN showed no significant difference. Therefore, 4 of 9 ZMYND8-associated downstream candidates were directionally correct, 3 showed no significant difference, and 2 changed in the opposite direction.

Quantitative summary of the validation results is shown in Fig. 3C. Among all 27 tested downstream genes, 10 genes were directionally concordant with the scGPA prediction, corresponding to an overall correct prediction rate of 37.0%; 14 genes (51.9%) showed no significant difference, and 3 genes (11.1%) changed in the opposite direction. When the analysis was restricted to genes with significant experimental changes, 10 of 13 genes were directionally concordant with the predicted direction, corresponding to a directional concordance of 76.9%. Stratified by target gene, the correct prediction rates among all tested candidates were 33.3% for IBSP, 33.3% for NPW and 44.4% for ZMYND8. These results indicate that scGPA can identify a subset of directionally responsive downstream genes after target-gene silencing. 

## Discussion

In this study, we developed scGPA to predict downstream transcriptional responses after target-gene silencing from single-cell transcriptomic data. The core idea is straightforward: a wild-type regulatory network is first inferred within a defined cell group, virtual knockout is then applied to the target gene, and the propagated perturbation is used to estimate the direction and magnitude of downstream transcriptional changes, followed by LLM-assisted organization and interpretation of the results. In experimental validation, scGPA achieved a prediction accuracy of 37.0% in osteosarcoma cells. These data indicate that scGPA can extract perturbation signals from single-cell data in a form that is directly testable in downstream experiments.

A key strength of scGPA is that it predicts not only which genes are affected, but also whether they are expected to increase or decrease after target-gene perturbation. Several representative methods can identify perturbed genes or regulatory programs, but do not directly provide gene-level directional outputs suitable for experimental follow-up. For example, D-SPIN is mainly designed to characterize perturbation-associated transcriptional states and gene programs [35], whereas GenKI focuses on identifying KO-responsive genes and their functional relevance [36]. These methods are informative for detecting responsive genes, but they do not naturally yield a clear up/down prediction for individual downstream targets. scGPA addresses this point by going beyond perturbation strength alone: after building the wild-type network, it attenuates the outgoing regulatory influence of the target gene and applies signed propagation to infer how the perturbation is transmitted through the network. As a result, the output contains both effect size and regulatory direction, which makes it more useful for direct validation by qRT-PCR and related assays. The consistency observed in osteosarcoma cells supports the practical value of this directional prediction strategy.

Unlike methods such as GEARS [37] and scGPT [31], which infer upregulation or downregulation from the difference between predicted post-perturbation and reference expression profiles, scGPA directly simulates perturbation-signal propagation within a cell-group-specific wild-type regulatory network. Methods based on predicted perturbed expression profiles generally require a comparable reference state and rely on consistent estimation of the expression scale for the perturbed and reference states. Consequently, common expression shifts between experimentally measured control and perturbed cells, as well as model-specific normalization and decoding procedures, may influence directional calling. scGPA does not require experimentally perturbed cells or post hoc subtraction between two independent expression profiles. Instead, it first infers a wild-type regulatory network within the user-selected cell group, attenuates the outgoing regulatory effects of the target gene, and propagates the resulting perturbation through signed regulatory edges. The sign of the accumulated propagation signal determines the final response direction of each downstream gene, directly linking the prediction to the transmission of reduced regulatory output from the perturbed target gene. To reduce the effects of cellular composition differences, measurement noise and sampling variability within the wild-type single-cell data, scGPA performs repeated subsampling within the same cell group and applies CP tensor decomposition to retain regulatory patterns that recur across subsamples, thereby obtaining a more stable wild-type regulatory matrix. Thus, scGPA converts direction calling from a reference-expression-dependent post hoc comparison into target-centered signed perturbation propagation within a cell-group-specific regulatory network. Its dose parameter also enables simulation of different degrees of gene silencing, making the model more compatible with partial knockdown experiments such as siRNA-mediated silencing.

A second advantage of scGPA is the use of an LLM as an interpretation and knowledge-integration layer. Large models are increasingly being introduced into single-cell analysis. For instance, Geneformer and scGPT are primarily used to learn transferable cell and gene representations for downstream tasks [17, 31], and scELMo further explores the use of LLM-based embeddings for target discovery and perturbation-related analysis [32]. In those settings, large models mainly function as representation learners or predictive modules. In scGPA, the role of the LLM is different: it does not replace the perturbation engine itself, but operates after the virtual knockout step to support cell-type annotation, candidate reranking, integration of database evidence and natural-language summarization. This division of labor preserves the mechanistic basis of the single-cell network model while making the final output easier to interpret and use in experimental work.

A third advantage of scGPA is that it combines single-cell preprocessing, virtual knockout inference and result interpretation within one workflow. For many wet-lab researchers, the practical bottleneck is not the lack of methods, but the fact that data preprocessing, clustering, cell-group selection, model execution and result curation are usually scattered across separate scripts and software environments. scGPA links these steps into a continuous pipeline: once the user provides the raw 10x data path, selects an LLM backend and enters the required API information, the system can return cell-group-specific virtual knockout results together with a ranked list of experimentally actionable candidate genes. This output helps researchers interpret candidate genes in two ways. On the one hand, the score and confidence value allow users to prioritize genes for experimental validation, rather than treating all predicted downstream genes equally. On the other hand, the evidence source and LLM-generated summary provide biological context, such as whether the candidate gene is functionally related to the perturbed gene, appears in relevant pathways or gene sets, or has interaction/literature evidence.

This study has some limitations. First, although scGPA achieved a higher overall correct prediction rate (23.0%) than GEARS (20.7%), scGPT (15.1%) and the empirical random baseline (13.6%), its predictive performance remains limited. Future work should improve its absolute predictive performance by refining regulatory network inference and uncertainty calibration and by integrating perturbation-training data and multi-omics information. Second, experimental validation was performed only in an osteosarcoma cell model, and further evaluation in additional tissues, cell types and disease contexts will be needed to assess the generalizability of scGPA more rigorously. Thirdly, the current framework relies only on transcriptomic data for prediction; future work should integrate multi-omics information, including proteomic, epigenomic and spatial data, to further improve the robustness and reliability of the predictions. Fourth, the current DoseDirKO perturbation rule uses a linear edge-scaling approximation to model target-gene silencing. Although this design provides an interpretable and computationally tractable way to attenuate the regulatory output of a target gene, it remains a simplified representation of biological regulation. In real cells, perturbation effects may be nonlinear, cell-context-dependent and influenced by feedback loops, compensatory pathways, post-transcriptional regulation and protein-level changes.

Overall, we developed scGPA, which features directional perturbation prediction, LLM-assisted interpretation and an integrated end-to-end workflow for single-cell analysis. Experimental validation showed that scGPA achieved a prediction accuracy of 37.0% in osteosarcoma cells. This work provides a new tool and methodological support for target-gene function inference and downstream mechanistic investigation based on single-cell transcriptomic data.

## Methods

### Overall framework

This study developed scGPA, an LLM-assisted virtual gene perturbation framework for single-cell transcriptomic data. The system couples two functional layers: a virtual-cell perturbation engine that estimates downstream transcriptional consequences of gene knockout within a given single-cell background, and a large-language-model (LLM) plus evidence-verification layer that refines, validates and explains candidate downstream genes.

### Single-cell data ingestion and preprocessing

scGPA accepts standard single-cell RNA-seq count matrices as input. For user-generated samples, raw sequencing data should first be processed by an upstream quantification workflow to generate the standard 10x Genomics-style files, including matrix.mtx(.gz), features.tsv or genes.tsv(.gz), and barcodes.tsv(.gz). For datasets downloaded from public repositories, users should ensure that the files are available in this format or convert them accordingly before running scGPA.

The system accepts either a single sample directory or a parent directory containing multiple 10x subdirectories. For multi-sample input, all valid 10x folders are recursively detected, and the matrices are merged in a union gene space while retaining sample identity by prefixing barcodes with sample-specific labels. This design enables subsequent explicit batch correction through sample-aware integration.

Before clustering, a standardized single-cell quality-control procedure is applied. For each cell, the system calculates n_genes_by_counts, total_counts, the percentage of mitochondrial transcripts (pct_counts_mt), and the percentage of ribosomal transcripts (pct_counts_ribo). Default filtering thresholds are n_genes_by_counts > = 200, n_genes_by_counts < = 8000, total_counts > = 500, pct_counts_mt < = 20%, and pct_counts_ribo < = 60%. Genes are further required to be detected in at least 3 cells. All quality-control thresholds and the retained numbers of cells and genes before and after filtering are exported to qc_summary.csv for reproducibility and auditing.

### Cell clustering and low-dimensional representation

After quality control, the expression matrix is normalized by total library size to a target sum of 1 × 10^4 per cell and subsequently transformed with log1p. The normalized matrix is stored in adata.raw for downstream marker detection. Highly variable genes (HVGs) are then identified, and by default the top 2,000 HVGs are retained for dimensionality reduction and clustering. The HVG matrix is scaled and subjected to principal component analysis (PCA).

For multi-sample input, Harmony integration is applied using the sample label to correct batch effects in PCA space; for single-sample input, the original PCA representation is used directly. A nearest-neighbor graph is then computed from the low-dimensional representation, followed by UMAP embedding and Leiden clustering with flavor = igraph, n_iterations = 2, and resolution = 0.5. To prevent dense-matrix expansion and memory overflow when handling large sparse matrices, sparse-friendly scaling and PCA implementations are used. Clustering outputs include cluster_annotations.csv, umap_coords.csv, umap_clusters_unannotated.png, and umap_clusters_annotated.png.

### Cluster marker discovery and marker filtering

For each cluster, differential expression is computed using the Wilcoxon rank-sum test. Marker genes are ranked by adjusted P value, nominal P value, and log fold change. All significantly upregulated markers satisfying *P* < 0.05 and log_2_FC > 0.5 are exported to cluster_markers_significant.csv for complete record keeping and manual review. After filtering, the top 100 genes per cluster are used for LLM-based annotation and saved to cluster_markers_top100_for_llm.json. A top-50 preview per cluster is also exported to cluster_markers_top50.json.

### LLM-assisted cell-type annotation

After clustering, the top marker genes of each cluster are then submitted to the LLM, which returns suggested cell-type labels in a structured JSON format. These labels are presented to the user as annotation suggestions. Users can either keep the LLM-suggested labels or manually provide customized labels based on canonical marker genes, prior biological knowledge or dataset-specific context. Thus, the LLM-assisted annotation step is designed to improve usability and reduce the manual burden of initial cell-group labeling, while retaining user control over the final cell-group selection.

### DoseDirKO-based virtual gene perturbation

DoseDirKO is based on the assumption that, within a selected cell group, the gene regulatory network inferred from wild-type scRNA-seq data can approximate the effective regulatory dependencies among genes. The DoseDirKO procedure consists of five main steps: inference of subsampled wild-type regulatory networks, CP tensor denoising to obtain a stable wild-type regulatory matrix, dose-aware attenuation of the target gene’s outgoing regulatory edges, signed perturbation propagation to estimate the response vector Δ, and Procrustes-aligned spectral embedding to calculate gene-level perturbation effect scores.

To obtain a robust wild-type regulatory matrix, DoseDirKO first infers multiple regulatory networks from repeated subsamples of the selected cell group. For each subsampling run b, a PCR/Ridge-based regulatory matrix $\:{\mathrm{W}}^{\mathrm{(}\mathrm{b}\mathrm{)}}$ is estimated, where $\:{\mathrm{W}}^{\mathrm{(}\mathrm{b}\mathrm{)}}\in{\mathrm{R}}^{\mathrm{G}\mathrm{x}\mathrm{G}}$ and $\:\mathrm{G}$ denotes the number of retained genes. The collection of $\:\mathrm{B}$ subsampled regulatory networks is then stacked into a third-order tensor:

$$\:\mathcal{T}\in\:{R}^{G\times\:G\times\:B}$$

where the first mode represents regulator genes, the second mode represents target genes and the third mode represents subsampling runs. CP decomposition is then used to approximate this tensor with a low-rank representation:

$$\:\underset{{\lambda\:}_{q},{a}_{q},{b}_{q},{c}_{q}}{\mathrm{min}}{\lVert\mathcal{T}-{\sum\:}_{q=1}^{Q}{\lambda\:}_{q}{a}_{q}\circ\:{b}_{q}\circ\:{c}_{q}\lVert}_{F}^{2}$$

where $\:\mathrm{Q}$ is the CP rank, $\:{{\uplambda\:}}_{\mathrm{q}}$ is the component weight, and $\:{\mathrm{a}}_{\mathrm{q}}$, $\:{\mathrm{b}}_{\mathrm{q}}$ and $\:{\mathrm{c}}_{\mathrm{q}}$ are latent factors corresponding to the regulator-gene, target-gene and subsampling modes, respectively. The denoised tensor $\:\widehat{\mathcal{T}}$ is reconstructed from the CP factors, and the final wild-type regulatory matrix is obtained by averaging the denoised networks across the subsampling mode:

$$\:{W}_{\mathrm{w}\mathrm{t}}=\frac{1}{B}{\sum\:}_{b=1}^{B}\widehat{\mathcal{T}}[:,:,b]$$

This step reduces unstable edge estimates caused by subsampling noise while retaining regulatory patterns that are consistently recovered across repeated subsamples.

In the denoised wild-type regulatory network, an edge from gene i to gene j represents the estimated regulatory influence of gene i on gene j in the local transcriptional state space. If a target gene is silenced or knocked out, its regulatory output to downstream genes is expected to decrease or disappear. Therefore, intervening on the outgoing edges of the target gene in the regulatory network provides a tractable approximation of the transcriptional consequences induced by target-gene perturbation.

For a target gene g0, DoseDirKO constructs a perturbed regulatory matrix by scaling the outgoing regulatory weights of g0:

$$\:{\mathrm{W}}_{\mathrm{k}}\mathrm{o}\mathrm{[}\mathrm{g}\mathrm{0,:]=(1-}\alpha\mathrm{)}{\mathrm{W}}_{\mathrm{w}}\mathrm{t}\mathrm{[}\mathrm{g}\mathrm{0,:]}$$

where $\:{\mathrm{W}}_{\mathrm{w}}\mathrm{t}$ denotes the inferred wild-type regulatory matrix, $\:{\mathrm{W}}_{\mathrm{k}}\mathrm{o}$ denotes the perturbed regulatory matrix and α denotes the perturbation strength. When α = 1, the outgoing regulatory effects of the target gene are completely removed, corresponding to complete virtual knockout. When 0 < α < 1, the model represents partial reduction of the target gene’s regulatory output, which is closer to transient knockdown experiments such as siRNA-mediated silencing. The signed transcriptional response was estimated by propagating the perturbation signal through the signed regulatory matrix. Let $\:{\overline{x}}_{{g}_{0}}$ denote the mean expression of the target gene g0 in the selected cell group. The initial perturbation input to gene j was defined as:

$$\:{u}_{j}=-\alpha\:{\overline{x}}_{{g}_{0}}{W}_{\mathrm{w}\mathrm{t}}[{g}_{0},j]$$

where $\:{u}_{j}$ represents the direct perturbation signal received by gene * j* from the attenuated target gene. The signed response vector Δ was then calculated by multi-hop propagation on the wild-type regulatory matrix:

$$\:\varDelta\:=u+{\sum\:}_{h=1}^{H}{\beta\:}^{h}({W}_{\mathrm{w}\mathrm{t}}^{T}{)}^{h}u$$

where * H* denotes the number of propagation steps and $\:\beta\:$ is the decay factor controlling the contribution of higher-order network effects. A positive $\:{\triangle}_{\mathrm{j}}$ indicates a predicted upregulation of gene $\:\mathrm{j}$ after target-gene perturbation, whereas a negative $\:{\triangle}_{\mathrm{j}}$ indicates a predicted downregulation. Across repeated runs, directional stability was summarized as:

$$\:{p}_{\mathrm{u}\mathrm{p},j}=\frac{1}{R}{\sum\:}_{r=1}^{R}I({\varDelta\:}_{j}^{\left(r\right)}>0)$$

$$\:{p}_{\mathrm{d}\mathrm{o}\mathrm{w}\mathrm{n},j}=\frac{1}{R}{\sum\:}_{r=1}^{R}I({\varDelta\:}_{j}^{\left(r\right)}<0)$$

where * R* is the number of repeated runs. These probabilities were used to define the directional confidence of each candidate gene.

To quantify the magnitude of the perturbation effect, DoseDirKO further compares the wild-type and perturbed regulatory networks in a node-level embedding space. The effect score is not calculated by directly comparing Wwt and Wko edge by edge. Instead, each signed and directed regulatory matrix W is first transformed into an unsigned symmetric similarity matrix:

$$\:S=\frac{\left|W\right|+|W{|}^{T}}{2}$$

This transformation is used only to estimate the magnitude of perturbation-induced network reorganization, whereas the direction of gene response is inferred separately from the signed regulatory propagation described above. We used this representation because scRNA-seq-inferred regulatory edges are sparse and noisy, and direct adjacency-matrix comparison mainly captures edge-level changes. In contrast, spectral embedding represents each gene according to its regulatory neighborhood in the graph, thereby providing a node-level representation that summarizes both local and higher-order network structure. Compared with graph diffusion-based measures, this approach avoids additional choices such as diffusion time, kernel scale or restart probability, which may introduce extra parameter sensitivity or over-smoothing. Other global network distance measures are useful for comparing whole networks, but they do not directly provide a gene-level effect score for candidate-gene ranking.

The normalized graph Laplacian is calculated as:

$$\:L=I-{D}^{-1/2}S{D}^{-1/2}$$

where * D* is the degree matrix of S. The low-dimensional embedding Z is obtained from the eigenvectors corresponding to the smallest non-trivial eigenvalues of L. Because spectral embeddings are identifiable only up to arbitrary rotation, reflection and sign changes, the knockout embedding is aligned to the wild-type embedding using orthogonal Procrustes alignment:

$$\:{R}^{\ast\:}=\underset{R}{\mathrm{a}\mathrm{r}\mathrm{g}\:\mathrm{m}\mathrm{i}\mathrm{n}}{\lVert{Z}_{\mathrm{w}\mathrm{t}}-{Z}_{\mathrm{k}\mathrm{o}}R\lVert}_{F}$$

$$\:{R}^{T}R=I$$

where * R* is an orthogonal rotation matrix satisfying $\:{\mathrm{R}}^{\mathrm{T}}\mathrm{R}\mathrm{=}\mathrm{I}$, and $\:{R}^{\ast\:}$ denotes the solution that minimizes the above Procrustes objective. The perturbation effect score for gene g is then defined as the Euclidean distance between its wild-type embedding and the aligned knockout embedding in the shared embedding space:

$$\:{E}_{g}={\lVert{z}_{\mathrm{w}\mathrm{t},g}-{z}_{\mathrm{k}\mathrm{o},g}{R}^{\ast\:}\lVert}_{2}$$

where $\:{E}_{g}$ denotes the perturbation effect score of gene g. Thus, the Procrustes-based effect score measures how strongly the regulatory network position of each gene is altered after target-gene perturbation. Genes with larger displacement are considered to have stronger perturbation-induced network repositioning and are prioritized as candidate downstream genes.

### Stability estimation and candidate compression

To reduce stochasticity induced by cell subsampling, the virtual knockout pipeline is repeated across multiple random seeds. For each gene, the model aggregates effect scores and direction responses across runs to obtain mean perturbation magnitude and directional stability. Genes are then separated into upregulated and downregulated candidate pools according to direction probabilities. During deployment, a restricted number of final candidates is shown in the user interface, but larger intermediate candidate pools are retained for evidence checking and LLM-assisted reranking.

### LLM-assisted candidate prioritization and evidence verification

After candidate compression, scGPA enters an LLM-assisted refinement stage for downstream candidate prioritization after virtual perturbation. This stage consists of four sequential steps: generation, evidence verification, modification and summarization.

First, in the generation stage, the LLM receives the target perturbation gene together with preliminary upregulated and downregulated candidate pools generated by DoseDirKO. Each candidate is accompanied by its perturbation-effect score and directional probability. The LLM returns an initial ordered list of candidate genes and short explanations for why each candidate gene may be relevant to target gene.

Second, in the evidence-verification stage, the system queries external biological knowledge sources to assess whether the candidate genes are biologically linked to the perturbed gene and whether evidence consistent with the predicted direction is available. Current evidence sources include NCBI Gene, PubMed, Gene Ontology, Reactome, KEGG, WikiPathways, MSigDB Hallmark, STRING, BioGRID and CORUM. In the current implementation, Gene Ontology, Reactome, KEGG, WikiPathways and MSigDB Hallmark are queried through Enrichr and g: Profiler; BioGRID evidence is used when a valid API key is provided, and CORUM evidence is used when a local CORUM file is configured.

Third, in the modification stage, the LLM receives the initial generation output together with the evidence-verification reports. Each evidence report contains the candidate gene, predicted direction, relationship-evidence score, direction-evidence score and representative evidence items retrieved from external biological resources. The LLM uses these structured reports to revise the preliminary upregulated and downregulated candidate lists. The numerical evidence scores are then incorporated into the final weighted ranking function:

$$\:{L}_{g}=1-\frac{{i}_{g}}{n}$$

where $\:{\mathrm{L}}_{\mathrm{g}}$ denotes the LLM-derived ranking score for candidate gene g, $\:{\mathrm{i}}_{\mathrm{g}}$ is the position of gene g in the LLM-revised list, starting from 0, and n is the number of genes in the revised list. Thus, genes ranked higher by the LLM receive larger LLM-derived scores.

The final candidate score integrates five components: perturbation strength from the virtual-cell model, model-derived directional probability, relationship evidence, directional evidence and the LLM-derived ranking score:

$$\:{S}_{g}=0.30{M}_{g}+0.20{D}_{g}+0.30{R}_{g}+0.10{V}_{g}+0.10{L}_{g}$$

where $\:{S}_{g}$ is the final ranking score of candidate gene g, $\:{M}_{g}$ is the normalized perturbation-effect strength from the virtual-cell model, $\:{D}_{g}$ is the model-derived directional probability, $\:{R}_{g}$ is the relationship-evidence score, $\:{V}_{g}$ is the directional-evidence score and $\:{L}_{g}$ is the LLM-derived ranking score.

Finally, in the summarization stage, the LLM generates a concise biological explanation of the most plausible upregulated and downregulated genes. The user-facing output includes ranked upregulated and downregulated genes, final scores, confidence values, representative evidence sources and a short natural-language summary.

To improve reproducibility, all LLM calls used structured JSON-only prompts and fixed temperature settings. The temperature was set to 0.0 for LLM-assisted cell-type annotation and 0.2 for LLM-assisted refinement and summarization. The final candidate ranking was not determined by free-form LLM output alone, but by a fixed weighted scoring function. If LLM generation, LLM modification or evidence retrieval failed, or if the returned content could not be parsed as valid JSON, scGPA fell back to the model-derived candidate ranking and continued execution.

### Implementation and output format

scGPA was implemented as a local-first workflow system with a guided startup workflow designed for users with limited coding experience. After downloading and extracting the package, users can launch scGPA by double-clicking the `Run-Agent.bat` file in the project folder (Fig. S1A). The startup script then opens a command-line interface that sequentially asks the user to provide the input scRNA-seq data folder, LLM provider, model name and API key (Fig. S1B). In the current implementation, the default LLM backend is DeepSeek with `deepseek-chat` as the default model. The framework also supports other models, including OpenAI and Gemini, and includes code-level support for local Ollama-based models. The LLM provider, base URL, model name and API key are configured by the user during startup.

After these inputs are provided, scGPA automatically starts the analysis workflow, including single-cell preprocessing, clustering, dimensionality reduction, batch correction when required and marker-gene identification. After clustering, scGPA uses the LLM to assist cell-type annotation. Marker genes calculated for each cluster are submitted to the LLM, which returns cell-group annotation suggestions in the command-line interface. Users can then select the cell group to be analyzed by keeping the LLM annotation or entering a customized label (Fig. S1C). After the target cell group is selected, scGPA launches a browser-based interface for virtual perturbation analysis.

In the web interface, users only need to enter the target gene for virtual knockout, such as `TP53` (Fig. S1D). scGPA then returns ranked predicted downstream genes in separate “Top Up” and “Top Down” lists. For each gene, the interface displays the final score, confidence value and representative evidence source. A concise LLM-generated summary is also shown below the ranked lists to provide a plain-language biological interpretation of the predicted downstream changes. Thus, the full workflow from data input to perturbation interpretation can be completed without writing code or manually inspecting intermediate computational files.

Figure S1A, scGPA can be launched locally by running the `Run-Agent.bat` script after downloading and extracting the package. Fig. S1B, During startup, users provide the input scRNA-seq data path, LLM provider, model name and API key. scGPA then automatically performs single-cell preprocessing and clustering. Fig. S1C, LLM-assisted cell-type annotation is generated from cluster marker genes, allowing users to select the cell group for virtual perturbation analysis. Fig. S1D, The web interface accepts a target gene, such as `TP53`, and returns ranked predicted upregulated and downregulated downstream genes together with scores, confidence values, evidence sources and an LLM-generated biological summary.

### Reproducibility and hyperparameter settings

To improve reproducibility, the main hyperparameters of scGPA were fixed unless otherwise specified by the user. In the default implementation, the number of subsampled networks was set to 15, the subsampling fraction to 0.8, the number of principal components for PCR-based network inference to 30, the Ridge regularization parameter to 1.0, the CP tensor rank to 5, the spectral embedding dimension to 20, the propagation decay factor β to 0.2, the number of propagation hops to 3, the number of repeated DoseDirKO runs to 20, the minimum cell fraction for gene filtering to 0.01, and the maximum number of retained highly variable genes to 2500. Candidate filtering used directional probability thresholds of 0.6 for both predicted upregulated and downregulated genes.

For CP/PARAFAC tensor denoising, the maximum number of iterations was set to 100, and the default TensorLy convergence tolerance of 1 × 10 − 8 based on absolute reconstruction error was used. Ridge regression was solved analytically and therefore did not require an iterative convergence criterion. Randomness from cell subsampling and PCA-based network construction was controlled by a user-defined random seed. In the default implementation, the random seed was set to 42, and repeated DoseDirKO runs used deterministic seed offsets from this initial seed. For LLM-related steps, structured JSON-only prompts were used. The temperature was set to 0.0 for LLM-assisted cell-type annotation and 0.2 for LLM-assisted candidate prioritization and summarization. If LLM generation, evidence retrieval or JSON parsing failed, scGPA fell back to the model-derived candidate ranking and continued execution.

For public Perturb-seq benchmarking, all parameters were fixed across datasets. The perturbation strength α was set to 1.0, the number of principal components to 50, the CP rank to 4, the embedding dimension to 16, the number of subsampled networks to 3, the number of repeated DoseDirKO runs to 3, and the random seed to 7. These reduced benchmark settings were used to ensure that all datasets and perturbation targets were evaluated under the same computational budget.

### Benchmarking on public Perturb-seq datasets

To benchmark the directional prediction performance of scGPA, we used five public Perturb-seq datasets: DixitRegev2016_K562_TFs_7_days [13],PapalexiSatija2021_eccite_RNA [22],NormanWeissman2019 [38],FrangiehIzar2021_RNA [9]andAdamsonWeissman2016_10 × 010 [39]. scGPA was compared with two representative perturbation-response prediction models, GEARS and scGPT.

Perturbation targets were initially screened using uniform data-quality criteria. A target was included in the initial candidate set when the following conditions were met: (i) the experimental condition represented a single-gene perturbation; (ii) at least 50 cells were available for the corresponding perturbation; (iii) the target gene was present in the expression matrix of the dataset; and (iv) the target gene was detected in at least five control cells. To ensure consistent comparisons among models, the analysis was further restricted to targets that could be jointly evaluated by scGPA, GEARS and scGPT within a harmonized input feature space.

For each perturbation target, each method generated a signed prediction score for candidate downstream genes. Genes with positive predicted scores were ranked as predicted upregulated genes, whereas genes with negative predicted scores were ranked as predicted downregulated genes. The top 10 predicted upregulated genes and top 10 predicted downregulated genes were retained for evaluation.

In addition, an empirical random gene-and-direction baseline was constructed under the same evaluation setting. For each perturbation target, 20 genes were sampled without replacement from the evaluable gene set of the corresponding dataset, excluding the perturbed target gene. Ten sampled genes were randomly assigned as predicted upregulated genes and the remaining ten as predicted downregulated genes. This procedure was repeated 1,000 times using a fixed random seed of 1007. In each repetition, predictions were classified as correct, opposite or showing no significant trend using the same experimentally observed labels and evaluation criteria applied to scGPA, GEARS and scGPT. The prediction counts and rates were averaged across the 1,000 repetitions for each target, and these target-level mean values were used for dataset-level summaries, pooled performance estimates and paired statistical comparisons.

For each dataset $\:\mathrm{(}\mathrm{d}\mathrm{)}$, perturbation target $\:\mathrm{(}\mathrm{t}\mathrm{)}$ and gene $\:\mathrm{(}\mathrm{g}\mathrm{)}$, the experimentally observed directional label was defined as:

$$\:{\mathrm{y}}_{\mathrm{d}\mathrm{,}\mathrm{t}\mathrm{,}\mathrm{g}}^{\mathrm{obs}}\mathrm{=}\left\{\begin{array}{c}\mathrm{1}\mathrm{,}{\triangle}_{\mathrm{d}\mathrm{,}\mathrm{t}\mathrm{,}\mathrm{g}}\mathrm{>}\tau\text{}\mathrm{a}\mathrm{n}\mathrm{d}\text{}{\mathrm{q}}_{\mathrm{d}\mathrm{,}\mathrm{t}\mathrm{,}\mathrm{g}}\mathrm{<}{\mathrm{q}}_{\mathrm{0}}\mathrm{,}\\\:\mathrm{-}\mathrm{1}\mathrm{,}{\triangle}_{\mathrm{d}\mathrm{,}\mathrm{t}\mathrm{,}\mathrm{g}}\mathrm{<}\mathrm{-}\tau\text{}\mathrm{a}\mathrm{n}\mathrm{d}\text{}{\mathrm{q}}_{\mathrm{d}\mathrm{,}\mathrm{t}\mathrm{,}\mathrm{g}}\mathrm{<}{\mathrm{q}}_{\mathrm{0}}\mathrm{,}\\\:\mathrm{0}\mathrm{,}\mathrm{o}\mathrm{t}\mathrm{h}\mathrm{e}\mathrm{r}\mathrm{w}\mathrm{i}\mathrm{s}\mathrm{e}\mathrm{,}\end{array}\right.$$

where $\:\mathrm{(}{\triangle}_{\mathrm{d}\mathrm{,}\mathrm{t}\mathrm{,}\mathrm{g}}\mathrm{)}$ denotes the difference in mean log-normalized expression between perturbed and control cells, and $\:\mathrm{(}{\mathrm{q}}_{\mathrm{d}\mathrm{,}\mathrm{t}\mathrm{,}\mathrm{g}}\mathrm{)}$ denotes the Benjamini–Hochberg-adjusted P value obtained from Welch’s t-test. The expression-difference threshold $\left(\tau\right)$ and FDR threshold $\:\mathrm{(}{\mathrm{q}}_{\mathrm{0}}\mathrm{)}$ were both set to 0.05.

For method$\:\mathrm{(}\mathrm{m}\mathrm{)}$, the predicted directional label was defined as:

$$\:{\widehat{\mathrm{y}}}_{\mathrm{d}\mathrm{,}\mathrm{t}\mathrm{,}\mathrm{m}\mathrm{,}\mathrm{g}}\mathrm{=}\left\{\begin{array}{c}\mathrm{1}\mathrm{,}\mathrm{g}\in{\mathrm{P}}_{\mathrm{d}\mathrm{,}\mathrm{t}\mathrm{,}\mathrm{m}}^{\mathrm{up}}\mathrm{,}\\\:\mathrm{-}\mathrm{1}\mathrm{,}\mathrm{g}\in{\mathrm{P}}_{\mathrm{d}\mathrm{,}\mathrm{t}\mathrm{,}\mathrm{m}}^{\mathrm{down}}\mathrm{,}\end{array}\right.$$

where $\:\mathrm{(}{\mathrm{P}}_{\mathrm{d}\mathrm{,}\mathrm{t}\mathrm{,}\mathrm{m}}^{\mathrm{up}}\mathrm{)}$ and $\:\mathrm{(}{\mathrm{P}}_{\mathrm{d}\mathrm{,}\mathrm{t}\mathrm{,}\mathrm{m}}^{\mathrm{down}}\mathrm{)}$ denote the top 10 predicted upregulated and downregulated genes, respectively. A prediction was classified as correct when its predicted direction matched a significant experimentally observed change:

$$\:{\mathrm{c}}_{\mathrm{d}\mathrm{,}\mathrm{t}\mathrm{,}\mathrm{m}\mathrm{,}\mathrm{g}}\mathrm{=}\mathrm{1}\left({\widehat{\mathrm{y}}}_{\mathrm{d}\mathrm{,}\mathrm{t}\mathrm{,}\mathrm{m}\mathrm{,}\mathrm{g}}\mathrm{=}{\mathrm{y}}_{\mathrm{d}\mathrm{,}\mathrm{t}\mathrm{,}\mathrm{g}}^{\mathrm{obs}}\neq{0}\right)$$

A prediction was classified as opposite when the observed direction was significant but opposite to the prediction, and as no trend when no significant experimental change was detected:

$$\:{\mathrm{o}}_{\mathrm{d}\mathrm{,}\mathrm{t}\mathrm{,}\mathrm{m}\mathrm{,}\mathrm{g}}\mathrm{=}\mathrm{1}\left({\widehat{\mathrm{y}}}_{\mathrm{d}\mathrm{,}\mathrm{t}\mathrm{,}\mathrm{m}\mathrm{,}\mathrm{g}}\mathrm{=-}{\mathrm{y}}_{\mathrm{d}\mathrm{,}\mathrm{t}\mathrm{,}\mathrm{g}}^{\mathrm{obs}}\neq0\right)\mathrm{,}$$

$$\:{\mathrm{n}}_{\mathrm{d}\mathrm{,}\mathrm{t}\mathrm{,}\mathrm{m}\mathrm{,}\mathrm{g}}\mathrm{=}\mathrm{1}\left({\mathrm{y}}_{\mathrm{d}\mathrm{,}\mathrm{t}\mathrm{,}\mathrm{g}}^{\mathrm{obs}}\mathrm{=0}\right)$$

The dataset-level correct prediction rate was calculated as:

$$\:Ac{c}_{d,m}=\frac{1}{20\left|{T}_{d}\right|}{\sum\:}_{t\in\:{T}_{d}}{\sum\:}_{g\in\:{P}_{d,t,m}^{up}\cup\:{P}_{d,t,m}^{down}}{c}_{d,t,m,g}$$

where $\mathrm{(}{\mathrm{T}}_{\mathrm{d}}\mathrm{)}$ denotes the set of perturbation targets retained in dataset $\mathrm{(}\mathrm{d}\mathrm{)}$. Because each target contributed 20 predictions, the total numbers of predictions in the five datasets were 180, 400, 1,280, 3,120 and 1,100, respectively.

Overall performance was calculated by pooling all retained targets:

$$\:Ac{c}_{m}=\frac{{\sum\:}_{d\in\:D}{\sum\:}_{t\in\:{T}_{d}}{\sum\:}_{g\in\:{P}_{d,t,m}^{up}\cup\:{P}_{d,t,m}^{down}}{c}_{d,t,m,g}}{20{\sum\:}_{d\in\:D}|{T}_{d}|}\:$$

where $\:\mathrm{(}\mathrm{D}\mathrm{)}$ denotes the five benchmark datasets. Because 304 targets were evaluated, the total denominator was 6,080 predictions for each method.

For statistical comparisons, a target-level correct prediction rate was calculated for each method:

$$\:{\mathrm{Acc}}_{\mathrm{d}\mathrm{,}\mathrm{t}\mathrm{,}\mathrm{m}}\mathrm{=}\frac{\mathrm{1}}{\mathrm{20}}{\sum\:}_{\mathrm{g}\in{\mathrm{P}}_{\mathrm{d}\mathrm{,}\mathrm{t}\mathrm{,}\mathrm{m}}^{\mathrm{up}}\cup{\mathrm{P}}_{\mathrm{d}\mathrm{,}\mathrm{t}\mathrm{,}\mathrm{m}}^{\mathrm{down}}}{\mathrm{c}}_{\mathrm{d}\mathrm{,}\mathrm{t}\mathrm{,}\mathrm{m}\mathrm{,}\mathrm{g}}$$

The target-level rates of scGPA were compared with those of GEARS, scGPT and the random baseline across the 304 matched perturbation targets using one-sided paired Wilcoxon signed-rank tests.

### Ablation analysis of the scGPA refinement modules

To distinguish the contribution of the core perturbation-prediction module from downstream evidence-guided and LLM-assisted refinement, we performed an ablation analysis using a fixed subset of 20 perturbation targets from four public Perturb-seq datasets, with five targets evaluated per dataset. The included targets were ELF1, ELK1, CREB1, EGR1 and GABPA from DixitRegev2016_K562_TFs_7_days; IFNGR1, ATF2, IRF1, JAK2 and NFKBIA from PapalexiSatija2021_eccite_RNA; BAK1, CEBPE, ETS2, KLF1 and UBASH3B from NormanWeissman2019; and ACTA2, AEBP1, B2M, CD274 and CD59 from FrangiehIzar2021_RNA. This secondary analysis was conducted separately from the all-target benchmark of 304 perturbation targets.

For each target, DoseDirKO first generated a fixed candidate pool consisting of the top predicted upregulated and downregulated genes. This candidate pool was kept identical across all ablation settings. The evidence-verification and LLM-refinement modules were allowed only to re-rank genes within this fixed candidate pool and were not allowed to introduce additional candidate genes.

Four variants were evaluated. First, DoseDirKO alone ranked candidate genes using only model-derived perturbation strength and directional probability. Second, DoseDirKO + evidence incorporated relationship-evidence and direction-evidence scores retrieved from external biological knowledge sources, but did not use LLM-derived ranking. Third, DoseDirKO + LLM incorporated the LLM-derived ranking score, but did not use external evidence scores. Fourth, the full scGPA pipeline integrated model-derived perturbation strength, directional probability, relationship evidence, direction evidence and LLM-derived ranking.

For each candidate gene g, the full scGPA score was calculated as:

$$\:{\mathrm{S}}_{\mathrm{g}}\mathrm{=0.30}{\mathrm{M}}_{\mathrm{g}}\mathrm{+0.20}{\mathrm{D}}_{\mathrm{g}}\mathrm{+0.30}{\mathrm{R}}_{\mathrm{g}}\mathrm{+0.10}{\mathrm{V}}_{\mathrm{g}}\mathrm{+0.10}{\mathrm{L}}_{\mathrm{g}}$$

where $\:{\mathrm{M}}_{\mathrm{g}}$ denotes the normalized model-derived perturbation strength, $\:{\mathrm{D}}_{\mathrm{g}}$ denotes the model-derived directional probability, $\:{\mathrm{R}}_{\mathrm{g}}$ denotes the relationship-evidence score, $\:{V}_{g}$ denotes the direction-evidence score and $\:{\mathrm{L}}_{\mathrm{g}}$ denotes the LLM-derived ranking score. For the ablated variants, only the retained components were used and their weights were renormalized to sum to one. Thus, DoseDirKO alone was scored as:

$$\:{\mathrm{S}}_{\mathrm{g}}\mathrm{=0.60}{\mathrm{M}}_{\mathrm{g}}\mathrm{+0.40}{\mathrm{D}}_{\mathrm{g}}$$

DoseDirKO + evidence was scored as:

$$\:{\mathrm{S}}_{\mathrm{g}}\mathrm{=0.333}{\mathrm{M}}_{\mathrm{g}}\mathrm{+0.222}{\mathrm{D}}_{\mathrm{g}}\mathrm{+0.333}{\mathrm{R}}_{\mathrm{g}}\mathrm{+0.111}{\mathrm{V}}_{\mathrm{g}}$$

DoseDirKO + LLM was scored as:

$$\:{\mathrm{S}}_{\mathrm{g}}\mathrm{=0.50}{\mathrm{M}}_{\mathrm{g}}\mathrm{+0.333}{\mathrm{D}}_{\mathrm{g}}\mathrm{+0.167}{\mathrm{L}}_{\mathrm{g}}$$

For each perturbation target and each ablation variant, the top five predicted upregulated and top five predicted downregulated genes were selected. Correct prediction rate was calculated as the proportion of selected genes whose predicted direction matched the experimentally observed significant transcriptional change in the Perturb-seq data. Dataset-level correct prediction rates were compared using paired Wilcoxon signed-rank tests.

### Reliability and robustness analysis

The score-stratified reliability, random-seed robustness and parameter-sensitivity analyses were performed using a fixed subset of 25 perturbation targets from five Perturb-seq datasets, with five targets evaluated per dataset. The subset comprised the 20 targets used in the ablation analysis together with ASCC3, DNAJC19, SCYL1, SEC61B and YIPF5 from AdamsonWeissman2016_10 × 010. For the score-stratified reliability analysis, each target contributed the top 10 predicted upregulated and top 10 predicted downregulated genes, resulting in 500 predictions. The same 25-target subset was used across all random-seed and parameter-sensitivity settings to ensure paired comparisons.

For each predicted gene, the signed perturbation score was calculated from the mean propagated response and its directional stability across repeated runs. The mean signed response of gene * g* was defined as:

$$\:{\overline{\varDelta\:}}_{g}=\frac{1}{R}{\sum\:}_{r=1}^{R}{\varDelta\:}_{g}^{\left(r\right)}$$

The signed perturbation score was then calculated as:

$$\:{Q}_{g}={\overline{\varDelta\:}}_{g}\underset{}{\mathrm{max}}\left({p}_{\mathrm{u}\mathrm{p},g},{p}_{\mathrm{d}\mathrm{o}\mathrm{w}\mathrm{n},g}\right)$$

where $\:{Q}_{g}$ denotes the signed perturbation score of gene * g*. A positive $\:{Q}_{g}$ indicates predicted upregulation, whereas a negative $\:{Q}_{g}$ indicates predicted downregulation. Its absolute value, $\:\left|{Q}_{g}\right|$, represents the model-derived effect magnitude. Predicted genes were ranked by $\:\left|{Q}_{g}\right|$ and divided into four quartiles.

To assess robustness to sampling variability, DoseDirKO was repeated using five different random seeds. For each perturbation target, the overlap of top-ranked predicted genes between two independent runs was quantified using the Jaccard index:

$$\:\mathrm{J}\mathrm{a}\mathrm{c}\mathrm{c}\mathrm{a}\mathrm{r}\mathrm{d}@K=\frac{|{A}_{K}\cap\:{B}_{K}|}{|{A}_{K}\cup\:{B}_{K}|}$$

$\:{\mathrm{A}}_{\mathrm{K}}$ and $\:{\mathrm{B}}_{\mathrm{K}}$denote the top-K predicted gene sets obtained from two independent runs, respectively. Directional concordance was calculated among overlapping genes as:

$$\:\mathrm{D}\mathrm{=}\frac{{\mathrm{N}}_{\mathrm{same}}}{{\mathrm{N}}_{\mathrm{overlap}}}$$

where $\:\mathrm{D}$ denotes directional concordance, $\:{\mathrm{N}}_{\mathrm{same}}$ denotes the number of overlapping genes assigned the same predicted direction in both runs, and $\:{\mathrm{N}}_{\mathrm{overlap}}$ denotes the total number of overlapping genes.

To evaluate parameter sensitivity, we varied three key parameters: perturbation strength (α = 0.5, 0.7 and 1.0), PCA dimensionality (pca_k = 30, 50 and 70) and CP tensor rank (cp_rank = 3, 4 and 5). For each parameter setting, the correct prediction rate was calculated. The stability of candidate-gene selection relative to the default parameter setting was calculated as:

$$\:{\mathrm{J}}_{\mathrm{default}}\mathrm{=}\frac{\mathrm{|}{\mathrm{A}}_{\mathrm{K}}\cap{\mathrm{B}}_{\mathrm{K}}\mathrm{|}}{\mathrm{|}{\mathrm{A}}_{\mathrm{K}}\cup{\mathrm{B}}_{\mathrm{K}}\mathrm{|}}$$

where $\:{\mathrm{A}}_{\mathrm{K}}$ denotes the top-K predicted gene set obtained using the default parameter setting, and $\:{\mathrm{B}}_{\mathrm{K}}$ denotes the top-K predicted gene set obtained using an alternative parameter setting. Directional concordance relative to the default parameter setting was calculated as:

$$\:{\mathrm{D}}_{\mathrm{default}}\mathrm{=}\frac{{\mathrm{N}}_{\mathrm{same,default}}}{{\mathrm{N}}_{\mathrm{overlap,default}}}$$

where $\:{\mathrm{N}}_{\mathrm{same,default}}$ default denotes the number of overlapping genes assigned the same predicted direction under the default and alternative parameter settings, and $\:{\mathrm{N}}_{\mathrm{overlap,default}}$ denotes the total number of overlapping genes between the two settings.

### Experimental design of scGPA predictions

To evaluate the predictive performance of scGPA, the publicly available osteosarcoma single-cell transcriptomic dataset GSE162454 was downloaded from the Gene Expression Omnibus (GEO) database. scGPA was applied to this dataset, and IBSP, NPW and ZMYND8 were selected as target genes for virtual knockdown analysis. The downstream genes predicted by scGPA are listed in Table 1 and were used for subsequent experimental validation.

### Cell culture and siRNA-mediated gene knockdown

The osteosarcoma validation experiment was performed using the 143B cell line (catalog no. H7-1001). For gene perturbation, siRNAs targeting IBSP, NPW or ZMYND8 were transfected into 143B osteosarcoma cells, and a non-targeting siRNA was used as the negative control (si-NC). The siRNA target sequences, including the si-NC sequence, are provided in Table S1. Cells were seeded 24 h before transfection. siRNA transfection was then performed, and total RNA was extracted 48 h after transfection. Knockdown efficiency was validated by qRT-PCR using GAPDH as the internal control. For each condition, three replicate wells were prepared, and the experiments were independently repeated three times.

### RNA extraction, reverse transcription and qRT-PCR

Following siRNA transfection, total RNA was extracted using a commercial RNA isolation kit according to the manufacturer’s instructions. cDNA was synthesized using a commercial reverse transcription kit. Genomic DNA removal was performed at 42 °C for 2 min, followed by reverse transcription in a final reaction volume of 20 µL under the following conditions: 37 °C for 15 min, 85 °C for 5 s and holding at 4 °C. The resulting cDNA was stored at -80 °C until use.

qRT-PCR was performed using a SYBR Green PCR kit on the QuantStudio platform. Each 10 µL reaction contained 5 µL SYBR Green, 0.5 µL forward primer, 0.5 µL reverse primer, 1.5 µL ddH2O and 2.5 µL cDNA. The cycling conditions were as follows: holding stage at 50 °C for 2 min and 95 °C for 10 min; amplification stage at 95 °C for 15 s and 60 °C for 1 min for 40 cycles; followed by melt curve analysis. Relative gene expression was calculated using the 2^-ΔΔCt method. Primers were synthesized by Sangon Biotech (Shanghai, China), with GAPDH used as the internal control. Detailed primer sequences are listed in Table S2.

## Supplementary Information

Supplementary Material 1.

Supplementary Material 2.

Supplementary Material 3.

## Data Availability

The osteosarcoma single-cell RNA-seq dataset analysed in this study is publicly available in the Gene Expression Omnibus (GEO) under accession code GSE162454 (https://www.ncbi.nlm.nih.gov/geo/query/acc.cgi?acc=GSE162454). The public Perturb-seq datasets used for benchmarking were accessed in harmonized form through the scPerturb collection (http://projects.sanderlab.org/scperturb/). The DixitRegev2016_K562_TFs_7_days, PapalexiSatija2021_eccite_RNA, NormanWeissman2019, FrangiehIzar2021_RNA and AdamsonWeissman2016_10 × 010 datasets were originally generated by Dixit et al., Papalexi et al., Norman et al., Frangieh et al. and Adamson et al., respectively. Additional data generated in this study are available from the corresponding author upon reasonable request.
