# Supplementary material for: scGPA: an LLM-assisted workflow for directional virtual gene perturbation analysis from single-cell transcriptomes
Source: BMC Genomics. 2026 Aug 25;27:765. doi: 10.1186/s12864-026-13270-0 (PMC13584509; doi:10.1186/s12864-026-13270-0)
Supplement: Supplementary file 1 [file 12864_2026_13270_MOESM1_ESM.docx]

**Table S1. siRNA sequences.**

| **Name** | **Sequence (5' to 3')** |
| --- | --- |
| si-IBSP-01-s | GCCUGUGCUUUCUCAAUGAAATT |
| si-IBSP-01-a | UUUCAUUGAGAAAGCACAGGCTT |
| si-IBSP-02-s | GAGACUUCAAAUGAAGGAGAATT |
| si-IBSP-02-a | UUCUCCUUCAUUUGAAGUCUCTT |
| si-IBSP-03-s | GAGAAGGAAAGUGAUGAAGAATT |
| si-IBSP-03-a | UUCUUCAUCACUUUCCUUCUCTT |
| hNPW-83-s | UGCCCUCCGGCGCGUGGUATT |
| hNPW-83-a | UACCACGCGCCGGAGGGCATT |
| hNPW-160-s | GGGCUGCGUCGCUCACCCUTT |
| hNPW-160-a | AGGGUGAGCGACGCAGCCCTT |
| hNPW-396-s | CAGCGGAGCUGGCCAGAGATT |
| hNPW-396-a | UCUCUGGCCAGCUCCGCUGTT |
| hZMYND8-2220-s | CGAACUGUCAUAGAUUUATT |
| hZMYND8-2220-a | UAAAUCUAUGACAAGUUCGTT |
| hZMYND8-1307-s | CCAAGAUCCUGAUGAGCAATT |
| hZMYND8-1307-a | UUGCUCAUCAGGAUCUUGGTT |
| hZMYND8-312-s | AGACCCUGUUGAUGUUGUATT |
| hZMYND8-312-a | UACAACAUCAACAGGGUCUTT |
| Negative Control-s | UUCUCCGAACGUGUCACGUTT |
| Negative Control-a | ACGUGACACGUUCGGAGAATT |
