# Supplementary material for: scGPA: an LLM-assisted workflow for directional virtual gene perturbation analysis from single-cell transcriptomes
Source: BMC Genomics. 2026 Aug 25;27:765. doi: 10.1186/s12864-026-13270-0 (PMC13584509; doi:10.1186/s12864-026-13270-0)
Supplement: Supplementary file 2 [file 12864_2026_13270_MOESM2_ESM.docx]

**Table S2. qRT-PCR primers used in this study**

| Gene | Forward primer (5'-3') | Reverse primer (5'-3') | Amplicon size (bp) |
| --- | --- | --- | --- |
| ACTB | AGGGGAGGTGATAGCATTGCT | GGCACGAAGGCTCATCATTCA | 111 |
| APOE | GGTCACATTCCTGGCAGGATG | AATCCCAAAAGCGACCCAGTG | 134 |
| B2M | GATGAGTATGCCTGCCGTGTG | TCATCCAATCCAAATGCGGCA | 119 |
| BASP1 | ATGCCAGCTTTCAGACAGAGC | CCCAACCACGGACATGCATTA | 162 |
| CCL5 | GAGGATCAAGACAGCACGTGG | GGTGTCCGAGGAATATGGGGA | 139 |
| CKB | CACTTCCTCTTCGACAAGCCC | TGTCATTGTGCCAGATACCGC | 94 |
| CPE | GGAATGAGGCTGTTGGACGAG | GGGTTCAGGGAAGGCATGATG | 133 |
| CSDE1 | AAAGAAACAAGCCCGCTGTCA | CATCGCCAGGCTGTAAGGTTT | 143 |
| CXCR4 | GTGGTCTATGTTGGCGTCTGG | CATTGGGGTAGAAGCGGTCAC | 115 |
| DDX5 | TCGGGTTATTCGAGTGACCGA | TCTCCCCAGGGTTTCCAAACT | 115 |
| FOS | CCGAGCTGGTGCATTACAGAG | GCGTTTTGCTACATCTCCGGA | 171 |
| GNG11 | TGCACAGAGCTACATCTCCCA | AGTGCAGAGGTCCAAGTGTCT | 95 |
| GZMA | CATCTGTGCTGGGGCTTTGAT | GTGAGCCCCAAGAATGACCTG | 94 |
| IBSP | AAGACACCACAGAGACCGGAA | ATTCATTGGCGCCCGTGTATT | 177 |
| IFITM5 | GACCACTTGATCTGGTCGGTG | TCTCGGGCCTTGATGGAGTAG | 92 |
| IGFBP5 | GTAAGTGCGCCAGACTCTTCC | CATGAAGGCCCAGCTTCTCTG | 126 |
| IGHG4 | GCATAGCAGGTCTACCCCAGA | CCTTTTGTGTGAGCACCTGGT | 116 |
| IGKC | ATAACGCCCTCCAATCGGGTA | GTAGTCTGCTTTGCTCAGCGT | 107 |
| JUN | GTCGACAAGTAAGAGTGCGGG | AGTTTCTCTAAGAGCGCACGC | 117 |
| LAPTM4B | GGGTGACATGCCTCGTATGTG | CTTAGTAAAAGCGGGCCCTGG | 116 |
| LGALS1 | GGGAACATCCTCCTGGACTCA | CGTTGAAGCGAGGGTTGAAGT | 178 |
| LUM | GCCCACTTCCCAAATCTCTGG | CCTCTTTCAGCCGATTGTGCT | 123 |
| NPW | CCAGAGACTTCGGAGAGACGT | TCTATGAGCGGCTCCTGGAAG | 171 |
| RAB2A | GCATGCTGCTACCAATGCAAC | TCATGTCTCAGCTGAGCAGGA | 153 |
| SDHC | TGGAGTGGTTGTCCTGGTTCT | GGAGAACTTTTCCCAGGCTGG | 153 |
| SELENOS | ACGGAAATCGGACAGAAAGCC | TTCTTAGCCTCATCCGCCAGA | 124 |
| SPARC | AGGTGTGCAGCAATGACAACA | GGATGTATTTGCAAGGCCCGA | 129 |
| TMEM119 | CCAGGATTAGGGATGTGGGGT | CTTCTAGGCTGCTTTGGAGGC | 157 |
| VAPA | GTGATCTCACCTCACTGCAGC | GCTCACACCTGTAATCCCAGC | 90 |
| ZMYND8 | CGATGCCCGATTCTTTGGACA | CTTCCTGCGGATGTTCTCCAC | 160 |
